# Composing Music Inspired by Sculpture: A Cross-Domain Mapping and Genetic Algorithm Approach

**DOI:** 10.3390/e24040468

**Published:** 2022-03-28

**Authors:** Francisco Braga, Helena Sofia Pinto

**Affiliations:** 1 INESC ID, 1000-029 Lisbon, Portugal; 2 Instituto Superior Técnico, 1049-001 Lisbon, Portugal

**Keywords:** computational creativity, inspiration, genetic algorithm, sculpture, musical composition

## Abstract

In this article, a system that takes a 3D model of a sculpture as starting point to compose music is presented. We raised the hypothesis that cross-domain mapping can be an approach to model inspiration. The semantic meaning of the sculpture is not used directly but rather a more abstract approach was used. A Genetic Algorithm was used to obtain results with more musical interest. The results were promising: the majority of the participants gave a classification of 4 out of 5 to the preferred interpretations of the compositions and related them to the respective sculpture. This is a step toward a possible model for inspiration.

## 1. Introduction and Motivation

Whenever we, humans, attempts to create something new, regardless of the field, we search for inspiration either from inside or from the world around us. Trash and Elliot consider two processes [1]: being inspired *by* and being inspired *to*. While being inspired *by* can be seen as the stimulus, being inspired *to* involves motivation. These two components are related with two important objects or ideas involved in the act of inspiration: the trigger, the stimulus object, associated with the “inspired *by*” process; and the target, the object to which the resulting motivation is targeted, associated with the “inspired *to*” process [1].

Existing ideas or objects inspire new ones in all sort of areas, from engineering, where solutions to new problems may be influenced by existing solutions to similar problems, to product design, where inspiration may come from shapes in nature [2], and art, where existing artworks can inspire one to create original art pieces [3]. Even though, in the above cases, inspiration comes from the same domain as the initial problem, it can also come from different domains. In the latter case, the challenge is usually more difficult because there is no direct connection.

Modeling inspiration computationally is far from being a reality. However, some systems address cross-domain inspiration as the mapping between two artifacts from different domains [4,5]. While Horn et al. mapped between two visual domains, images into 3D-printable ceramic vases [4], Teixeira and Pinto mapped images into sounds [5] involving two different domains. The basis for this mapping uses as a starting point an attempt to find and model the essence of an already existing artifact, and one of the main challenges of the approach is how to relate two different domains.

This article describes our effort in creating a system that composes music inspired by sculptures. We decided to draw inspiration from existing human-made artworks to maintain the human hand in the equation. The features used to characterize both domains and their respective mapping between the sculpture and music domains were guided by the aesthetics of the authors. Other features and possible mappings could have been explored. Although the approach described here is only one of an infinite number of possibilities, based on our evaluation results, it was successful. Three goals have driven our research:

- Build a system that acts both creatively and generates a product that is considered creative.
- The music pieces composed by the system are inspired by the sculpture; therefore, in a certain way, the music is associated with the sculpture.
- Build a system that composes music deemed aesthetically pleasing.

In the next section, related work is described. Then, our approach to sculpture inspired musical composition is explained, starting by describing the representation, shape, and the texture features that were considered. The mapping and composer approach to music composition that was implemented is also described. Section Case Studies describes how our listeners perceived the composed music. Then, a discussion on our results and future directions concludes the paper.

## 2. Related Work

Our work focuses on two main areas: the generation of musical compositions and the act of inspiration. Regarding the generation of musical compositions, there is relevant work both in the algorithmic composition and in the computational creativity fields. As to inspiration, this is still an under-explored topic [4].

### 2.1. Generation of Musical Compositions

Algorithmic composition can be described as *“the process of using some formal process to make music with minimal human intervention”* [6]. The simplest form of algorithmic composition lies in randomness. Many different examples can be inserted here, such as the technique attributed to composer Wolfgang Amadeus Mozart, called *Musikalisches Würfelspiel* (“musical dice game”) [6], which consists of using a dice to combine already existing musical fragments.

There are some examples where the music is not made entirely by chance, but still, there is some randomness associated with it. One good example, is John Cage’s composition named *Reunion* [6]. It consists in playing chess in a photo-receptor equipped chessboard, and for a certain move, a sound would be triggered, thus creating a different piece for each performance.

There are also some examples of more complex rule-based algorithmic composition, such as the Twelve-tone technique [7], invented by the Austrian composer Arnold Schoenberg. This technique ensures the use of all twelve notes of the chromatic scale, and that all of them are equal. As such, each note can only be repeated once all the others have already been played. The composition process begins with the creation of a tone row, consisting in an ordered row of all twelve notes. The entire piece will then consist in the repetition of this row, as many times as desired. To make the music more interesting, several transformations can then be applied to this row in the following repetitions. Many composers used pre-composed series, thus, creating music based on the rules of the twelve-tone technique.

In the computational realm, some systems combine randomness with rules. The first computer-generated composition [8] was made by Lejaren Hiller and Leonard Isaacson at the end of the 1950s using Illiac (Illinois Automatic Computer). The algorithm starts generating random integers that represent musical elements, such as pitch, dynamics, and duration. After this, the obtained values are validated according to rules where the values that fail are re-generated. This paradigm of generator/modifier/selector used in this project was imperative and was even later used in systems, such as Musicomp [9].

In the field of computational creativity, several artificial intelligence methods have been used to address this goal, from Mathematical Models to Learning Algorithms [10]. The two methods that are more relevant to our work are knowledge-based systems and evolutionary methods.

An example of using a knowledge-based system was developed in the 1980’s by composer David Cope, that later named it EMI (Experiments in Music Intelligence) [11]. The system aimed at creating new pieces of music based on a specific style or author. Cope’s approach considers that a musical phrase can be analyzed as a natural language phrase, i.e., musical functions can be matched to parts of the speech. distributed by the remaining non-zero probabilities. One issue with this approach is that, Based on this idea, new examples of music could easily be composed. As such, the system analyzes a group of musical pieces and attributes identifiers to notes. With this, a signature dictionary is created where these signatures are what characterizes a composer or style, making it unique. Afterwards a lexicon is built considering the previous steps. Music is then created using a musical augmented transitional network (ATN).

Concerning evolutionary methods, GenJam [12] is a system that generates jazz solos given a certain chord progression. The system proposed by Biles solves the jazz solo search problem using a genetic algorithm. Variations [13] is a system that applies a genetic algorithm to the composition process with as few restrictions as possible. To do so, the author attempted to describe, with as few rules as possible, the composition process and convert it into a computer program.

It is relevant to note that, although the complex rule-based approach found on EMI was very successful in creating pieces in a specific style [11], systems that use genetic algorithms are the ones most associated with novelty. In the latter, the level of constraints imposed by these rules will change the final result drastically. In the system Variations [13], the author uses as few rules as possible, leading to the composition of a not-so-well-defined piece of music with very few structural elements. On the other hand, considering GenJam [12], Biles imposes many rules and constraints, leading to more structured results, as intended, but lacking in novelty.

### 2.2. Inspirational Systems

Although inspiration is a complex topic, there are some examples of research on inspirational systems in the field of computational creativity. Horn et al. [4] proposed a cross-domain inspiration framework with the system Visual Information Vases (VIV). In this system, the authors consider cross-domain inspiration to be a cross-domain analogy mapping. Using this model for inspiration, the system produces 3D printable vases inspired by 2D images provided by the user. The image is processed, and using the obtained color palette from dominant and salient colors, the mapping to 3D printable vases is made considering four aesthetic measures: activity, warmth, weight, and hardness. VIV then uses a genetic algorithm to produce a vase with an aesthetic profile close to the one from the image’s color palette.

Teixeira and Pinto used a similar cross-domain inspiration framework in their system Cross-Domain Analogy: From Image to Music [5]. This system generates a piece of music inspired by a given image. By considering a similar inspiration model as the one previously described, this system processes the image, and from the extracted features attempts to map the emotional mood present in the image to the musical artifact. Two artifacts are produced from the mapping, one with very few rules and one with more constraints. The authors then use a Genetic Algorithm to evolve these two artifacts, thus, obtaining a balance between them. The algorithm does not impose more rules but favors the most musically pleasing artifacts as guided by the fitness function.

## 3. Sculpture Inspired Musical Composition

**Figure 1:**
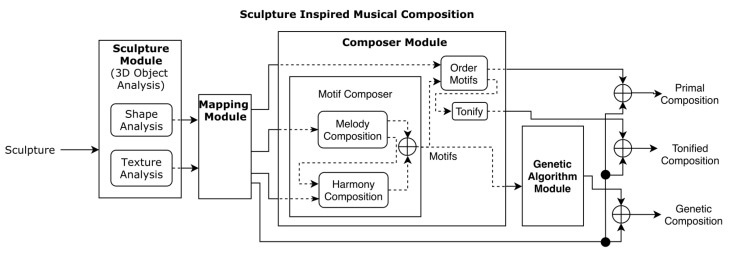
System architecture.

In our system, a model for inspiration similar to the inspirational systems previously described is used. Regarding musical composition, a balance is intended between randomness and rules regarding the mapping from sculpture features to musical elements. A genetic algorithm was also used with this in mind. A diagram of our system’s architecture can be seen in Figure 1. Below, each module of our system is explained.

### 3.1. Sculpture Module

When first looking at a sculpture, several characteristics come to our attention. These can be divided into two groups: shape features, regarding the shape of the sculpture, and texture features, regarding the color or texture of the sculpture. We decided not to address the semantic meaning of sculptures because this would be a complex task both computationally and in terms of interpretation, since each individual may have a different perception of a sculpture. Thus, a more general approach that does not address semantics was chosen, leading to a more abstract interpretation.

#### 3.1.1. Representation

There are many possibilities to obtain a computational representation of a sculpture, such as retrieving it from images; however, the one followed by us was using 3D objects since all the information regarding shape and texture can be more easily obtained.

There are several different possible representations of the 3D objects. Polygonal Meshes [14] were used, since this is a simple representation, yet it has all the needed information. In this representation, the object is represented by its boundary surface, composed of several planar shapes, which are defined by a series of vertices connected by edges. In our work, the most commonly used shape was used, the triangle (triangular meshes).

**Figure 2:**
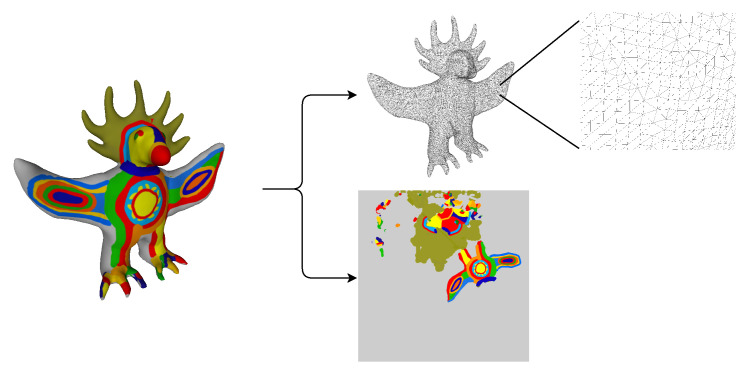
Example 3D mesh with texture.

Despite being 3D objects, when it comes to the texture, it refers to a 2D surface. A simple analogy is a gift wrap. The gift itself is a 3D object, but the wrapping alone is a 2D surface. To do the mapping of the texture in the 3D object to 2D, a mapping is used, that takes the coordinates of a 3D object, *(x,y,z)*, and converts them to 2D coordinates, *(U,V)*. A visual example of the representation can be seen in Figure 2.

#### 3.1.2. Shape Features

Considering shape features, three main features were extracted: curvature, angles, and segments. There are many possibilities to quantify the curvature of a given surface. From all the available options, the mean curvature [15] was chosen, where each vertex of the mesh has its mean curvature value.

Regarding the angles, these are measured using the normal vector of the faces. To obtain each vertex’s angle, the angles between all its faces are measured, and only the maximum is considered. One important aspect to note is that, since the angle is obtained from the normal vector, if the faces form a plane surface, the angle is 0°, while two overlapping faces have an angle of 180°. Using the angles, another measure was obtained, which was named zero angle predominance. This measure verifies if the vertices with 0° angles in an object are inserted in a plane surface, or if they occur separately. If they are inserted in a plane surface, then our focus goes towards what arises from it, or to the limits of that surface. To obtain this measure, first, the number of vertices that have an angle of 0° is obtained. Then, from those, it is verified how many also have at least half of their neighbors with an angle of 0°. The zero angle predominance is the ratio of the second to the first.

**Figure 3:**
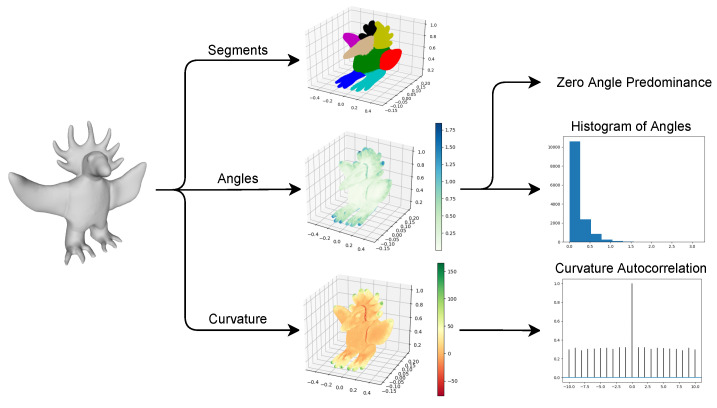
Example of extracted shape features.

Finally, the segmentation is performed using the spectral clustering algorithm [16]. This algorithm fits our purpose since its roots are on graph theory, and a mesh can be seen as a graph. It receives the adjacency matrix of the object’s vertices and the number of clusters, returning a label for each vertex. Using this, the mesh was divided into segments. A fixed number of eight clusters was used; however, this number could be changed from sculpture to sculpture if desired. An example of the three shape features can be seen in Figure 3.

#### 3.1.3. Texture Features

Since the texture is a 2D image, the feature-extraction process is performed using the same feature extraction algorithms as in a 2D image. Considering color representation, the ones more relevant to us are RGB and HSV. On the one hand, in RGB, any color is described as a combination of values of the three main additive colors: Red, Green, and Blue.

On the other hand, HSV refers to a combination of Hue, Saturation, and Value. Hue refers to the color itself, saturation to the intensity (purity) of a particular hue, and value to the lightness or darkness of that color. Both models were used for different purposes.

There are several different color features. The two main categories histogram-based methods and color statistics [17]. Color histogram-based methods mainly represent the distribution of colors in an image. Although there are several ways to do this, the most common and useful way is the histogram of the distribution over the color model (3D in the case of RGB and HSV) or each channel of the color model. Color Statistics are statistical measures, such as the value, standard deviation, median, and percentiles, among others. Aside from these two categories, several features can be obtained by processing the image. From those, the two extracted and used in our work are:

- Most Common Colors—To obtain these colors, a simple counting approach was used. First, nine equally spaced values were defined for each channel of the RGB model (thus, a total of 729 distinct colors). Having the image’s pixels represented in the same model, for each pixel, the closest value for each channel is chosen, and that color’s count is increased.
- Perceived Brightness—This can be seen as a conversion from the original colors to a greyscale based on the brightness of each color. In our work, a formula where different brightness weights are attributed to each of the RGB channels was used [18]: $PB=\sqrt{0.299*R^{2}+0.587*G^{2}+0.114*B^{2}}$

### 3.2. Mapping and Composer Module

Since our aim is to compose music inspired by sculpture, an analogy from one domain to the other was made. However, since music is such a vast field, it becomes quite impossible not to restrict. As such, we decided to focus our approach on modal music since, with modal harmony, one can more easily pass on a specific sensation, associated with the respective mode. In addition to this, only the most common time signature in Western music, ${}_{4}^{4}$, was used.

The first step towards creating the analogy was to map the sculpture’s features into musical elements and concepts. As a starting point, it was not our aim to take an overly precise approach, for example, where each point would be converted into a note. We intended to obtain the musical elements through the analysis of the sculpture’s features in a more general manner, by considering either the sculpture or each of the sculpture’s segments as a whole. With this in mind, we start with a high-level association between the two groups of a sculpture’s features, shape and texture, and the considered elements of a musical piece: melody, harmony, and rhythm.

If a melody is listened to without any harmony (chords), one may perceive a particular sensation or emotion. Once it is listened to with harmony, this sensation or emotion may vary drastically from the initial one. Moving to the sculpture domain, considering a sculpture’s shape, mainly sensations associated with its characteristics are obtained, such as smoothness or roughness. However, once added the texture/color, once again, a much better-defined sensation, or even emotion, is obtained. One can say that the same way texture/color gives context to the shape, harmony gives context to the melody. As such, our approach’s foundations lie in the association of the sculpture’s shape with melody, and the texture/color with harmony. This is not strict, and some associations were made from texture to melody. There is still one part of the music piece left to map: the rhythm.

As already stated, the rhythm is associated with the time signature and the tempo. Concerning the time signature, as already stated, only the most common time signature in western music, ${}_{4}^{4}$, was used. Although initially both shape and texture were considered for the tempo, only the texture was used. This decision was made when confronted with the final results, which shall be later explained.

Regarding the composition, motifs were used. A motif can be described as a short musical idea that often occurs in a piece of music. It does not need to be always equal, and may be subject to changes while maintaining its general idea, thus, providing the music piece a sense of unity. As explained before, a sculpture can be segmented. As such, we decided to compose each motif using features obtained from each segment. To obtain the size of the motif, each segment’s size in relation to the whole sculpture was used.

#### 3.2.1. Motif Composition—Melody

Starting with the melody, this element consists of a linear sequence of notes, while each note consists of pitch and duration. Accordingly, we sought to associate these two characteristics with shape characteristics.

The pitch of the notes was obtained using the sculpture’s lines or curves. If the lines of the sculpture are smooth, the pitches of the melody should transmit the same sensation, as well as the contrary. To obtain the line’s description, the angles were used. Using the angles histogram, the musical interval for the next note concerning the current note was related to each *bin* of the histogram. The interval between pitches is measured by tones, where the smallest interval in usually found in western music is the semitone. In Table 1, the common names given to intervals can be found. These are given according to the number of semitones that separate two pitches.

Since, in one octave, there are twelve possible intervals, the histogram was calculated using twelve *bins*. With the histogram, a probability distribution to obtain a musical interval was created, thus, having the probability for the next note’s interval concerning the current note. The mapping made from angles to musical intervals can be seen in Table 2.

At this point, the Zero Angle Predominance feature was used. As explained before, this feature identifies if a sculpture’s angles of 0° are an important element, or if they correspond to a plane. If they correspond to a plane, the protrusions or objects that arise from that plane or the angle between this and another plane should matter the most. As such, in this case, the probability for the interval associated with the angles near 0° should be reduced in favor of the others. To do so, we set the maximum cut for the probability of the interval associated with the 0° at 80%. Then, from the zero angle predominance ratio, we obtained how much the cut is by mapping the ratio (from 0 to 1) to the cut for probability (from 0% to 80%). What has been cut is then equally distributed by the remaining bins that do not have a probability of zero.

One issue with this approach is that, if one particular *bin* does not contain any occurrences, that interval will never be used. We acknowledged that an interval of angles could not only be mapped to the respective musical interval but also the neighbors’ musical intervals considering the order of Table 2. With this in mind, after obtaining the musical interval, a normal distribution is also applied, to give the neighboring musical intervals a chance to be chosen.

The standard normal distribution was used (μ=0 and σ=1). Using a value obtained from the normal distribution, there are three possible cases: if the value is between −1 and 1 (probability of 68.3%), the original musical interval is chosen; If the value is below −1 (probability of 15.85%), the left neighbor musical interval is chosen, unless the original value is the first position, in this case, the original musical interval is chosen; If the value is above 1 (probability of 15.85%), the right neighbor musical interval is chosen, unless the original value is the last position, in this case, the original musical interval is chosen.

The only aspect left to decide is the direction of the interval, which is the only musical element in the melody mapped from the texture. Here, we decided to use of the perceived brightness. Colors with a higher perceived brightness value are related to upward intervals, and colors with lower values with downward intervals. From the histogram of perceived brightness values, the value that occurred more often was considered. This ranges from 0 to 1 and sets the probability of the interval direction being upward between 20% and 80%.

**Figure 4:**
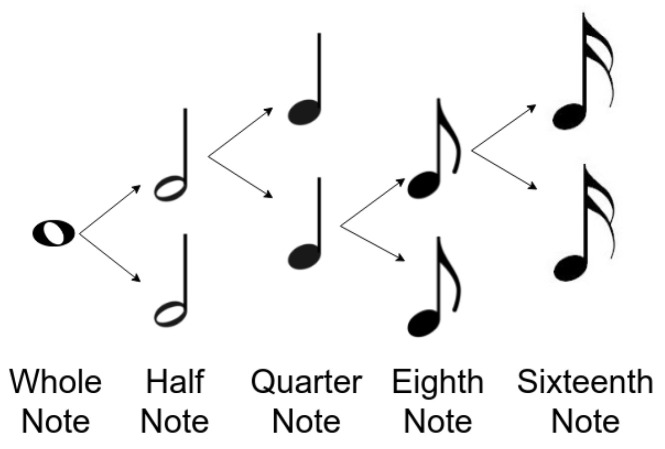
Representation of note durations.

The duration of a note is the time the note lasts. This will significantly influence the way a music piece is perceived. This is represented as a fraction of what is called a whole note. For our purposes, not all possible durations were used. The mentioned representation and the considered durations can be seen in Figure 4.

If a sculpture with an irregular surface curvature value is considered, one can easily associate it with a dense music piece, where the notes keep changing in a small fragment of time. The opposite can also be related. As such, our aim was to relate the variation of the sculpture’s curvature value with the note’s duration. To obtain the curvature value variation, autocorrelation was used. The curvature value of each point was gathered, grouped by neighbors, and the autocorrelation was calculated in these groups. The mean of all results was used, and this value was mapped with what would become the most probable rhythmic value. The mapping can be seen in Table 3.

Our goal was to avoid having only one type of rhythmic value. Our goal was to find a general way to relate the mean autocorrelation with the rhythmic value without making a direct relation. To achieve this, the Standard Normal Distribution was used once again. Using this tool, the most probable rhythmic value can be set while maintaining the possibility of using other rhythmic values. For each note, a rhythmic value is chosen based on the most probable one and a value obtained using the normal distribution (Table 4).

#### 3.2.2. Motif Composition—Harmony

Having the various motifs that will form the melody, let us move on to harmony. We decided to relate the harmony with the sculpture’s texture/color. As such, each motif should be harmonized according to the respective segment’s color. For each segment, the most common colors were obtained, as explained before. Having the HSV color model in mind, for the same Hue and the highest Saturation (100%), the Value channel will give us how dark or how pure that Hue is, ranging from black (Value close to 0%) to the pure Hue (Value close to 100%). Similarly, the musical modes can also be ordered from darker (Locrian) to brighter (Lydian) [19]. In Table 5, a summary of the modes of the major scale is shown, with the degrees of the major scale, the respective mode, intervals, and chord.

Having the most common colors, the Value channel from the HSV color model was related to musical modes. A color with a lower value in the Value channel would be associated with a darker mode, while one with a higher value with a brighter mode. However, not all Hues should be matched with all modes, since, for the same Value, some colors are perceived as darker than others.

**Figure 5:**
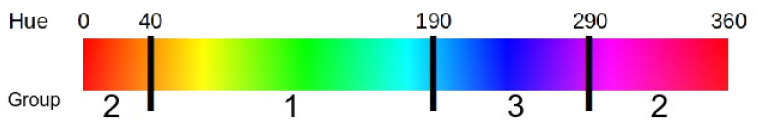
Hue value based groups.

Three groups of Hues were formed, as seen in Figure 5:

- Group 1 (Hue from 40 to 190), which includes colors, such as Yellow, Green, and Light Blue, where the modes can range from Lydian to Dorian;
- Group 2 (Hue from 0 to 40 and from 290 to 360), which includes colors, such as Red, Pink, and Magenta, where the modes can range from Ionian to Aeolian;
- Group 3 (Hue from 190 to 190), which includes colors, such as Purple, and Blue, where the modes can range from Mixolydian to Phrygian.

In addition to these three groups, two special cases are not accounted for by the Hue values:

- If the color is black, or very close to black (Value channel below 20%), the mode is set to Locrian (the darkest).
- If the color is white, or very close to white (Saturation channel below 10%), the mode is set to Lydian (the brightest).

**Figure 6:**
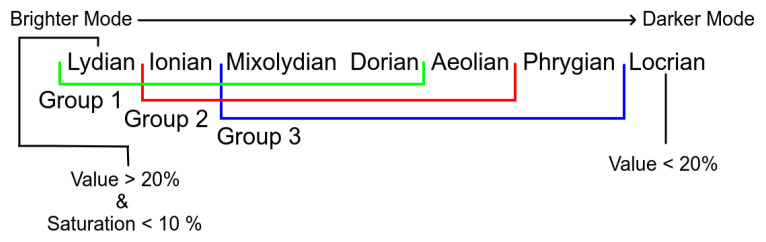
Range of modes per color group and special cases.

Even if the Saturation is near 0%. if the Value channel is close to 0%, the color will be close to black. As such, the first verification to be made is if the color is close to black (Value < 20%). The range of modes per color group association and the special cases can be seen in Figure 6.

**Figure 7:**
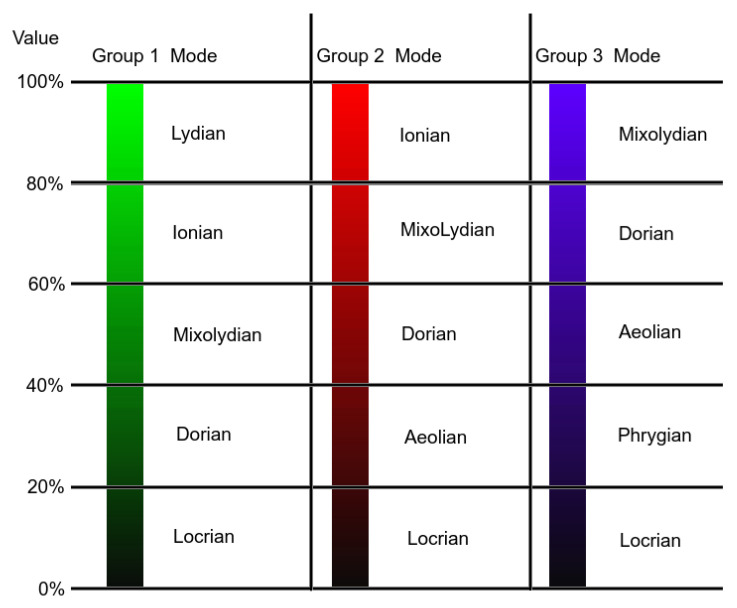
Relation of Hue-based groups with modes according to the Value channel.

The relationship of the Hue-based groups with the modes according to the Value channel can be seen in Figure 7, where, for each group, an example of a Hue is shown.

Having associated musical modes with colors, the chords still needed to be defined. These were obtained by finding the closest tone of each motif and then obtaining the mode’s chord in that tonality, as seen in Table 5. One aspect that was left to decide was the number of chords per motif.

First, we decided that there should be, at most, one chord per bar, since there should be enough time for the mode to be well perceived. Moreover, we decided to use at most two chords per motif for the same reason. As such, for each segment, the two most common colors are extracted, from there the two respective modes, and finally, the two chords. Considering that the first half of a motif is stronger than the second half, the chord obtained from the most common color is placed first in the motif and the one from the second most common chord second. The number of bars for the first chord is obtained by dividing the motif’s number of bars by two and rounding up, while the remaining bars are left for the second chord. Having a set of different motifs and their respective harmony, their order and the tempo stills needed to be defined.

### 3.3. Order Motifs and Tempo

To join the motifs in a logical order, the probability distribution of intervals from the histogram of angles was obtained, although this time from the whole sculpture. From there, we verified which of all possible permutations of the motifs’ order best corresponds to the probability distribution of the intervals—this is the chosen order.

The only musical element left to map is the tempo. At first, our aim was to map this element both from the shape and the texture of the sculpture. It made sense to us that it should be related to the variation of both shape and texture. However, while experimenting, we realized that mixing both types of features was not the best choice, since the result had the possibility of not being associated with the sculpture. For example, let us consider a sculpture with a very smooth shape that does not vary much, while in terms of texture, there is much variation. Our first approach would lead us to a medium tempo since it would be a mix of not much variation from the shape and much variation from the texture. As such, since the shape’s variation had already been used for the rhythmic value of notes, the texture’s variation was used for the tempo, obtaining much better results. To find the variation of the texture, first, the most common colors of the whole sculpture were extracted. Then, for each segment, we verified how many of the most common colors of that segment matched the ones from the whole sculpture. In the end, a ratio of the colors that matched the total colors verified was obtained, that ranges from 0 to 1. The higher the ratio, the lower the tempo should be, and vice versa. For our purposes, we considered a tempo range of 80 to 160 BPM. As such, having the ratio (*r*), the tempo is calculated using tempo=80+80 ∗ (1−r).

Having the melody and harmony from the ordered motifs, and respective tempo, the first version of the music piece was obtained, which was designated as the raw composition. The second version was obtained by correcting notes in the motifs that did not belong to the respective scale, changing them to the closest note found on the scale. This way, a version that respects each motif’s scale was obtained, designated as the “tonified” composition. We also decided to use a Genetic Algorithm, since it provides a way to search for better results while retaining some randomness that may lead to exciting results.

### 3.4. Genetic Algorithm

A Genetic Algorithm is a search algorithm inspired by the process of natural evolution first introduced by Holland et al. [20]. This type of algorithm has proven to be very effective in dealing with large search spaces. To perform the search, nature-inspired mechanisms are used.

In a genetic algorithm, there is a population that consists of a set of what are called individuals. Each individual is a candidate solution and has a set of genetic information, called genes. These individuals are evolved in order to find the best possible solution. The number of individuals in the population is one of the features of this algorithm. Different representations can be used for individuals, depending on the use of the algorithm.

The first step in a genetic algorithm is to set the initial population, which may be randomly created or not. In order to evaluate each candidate’s solution, there is a fitness function that rates each individual. The evolutionary phase of the algorithm consists of a specific number of iterations, where in each iteration, a new set of individuals is generated. Thus, for each iteration, a new generation arises.

Each new generation is created using the previously mentioned nature-inspired mechanisms, called genetic operators. There are three main genetic operators: selection, crossover, and mutation. In addition to these three operators, there is another commonly used mechanism, named elitism. Furthermore, the probability associated with each genetic operator is independent of the other operators. As such, a particular genetic operator may or may not be applied regardless of the others. Each new generation is created according to the following steps:

1. **Elitism—**Select a part of the individuals according to their fitness to be directly passed to the new generation.
2. **Selection—**Individuals are selected to breed the new generation. The selection occurs in pairs, where each pair of individuals produce new individuals for the new generation. The probability of each individual to be chosen is based on the individual’s fitness. The higher the fitness, the higher the probability of being chosen. As such, one individual may be selected several times or none at all.
3. **Crossover—**Having a pair of selected individuals, there is a certain probability of suffering crossover. The crossover mechanism is much like natural reproduction, where genetic information (genes) from both parents (individuals) is combined to generate new ones.
4. **Mutation—**Afterwards, the individuals obtained have a probability of being mutated. This involves altering the individual’s genes. This genetic operator can either be applied to each gene separately or to all genes.

**Figure 8:**
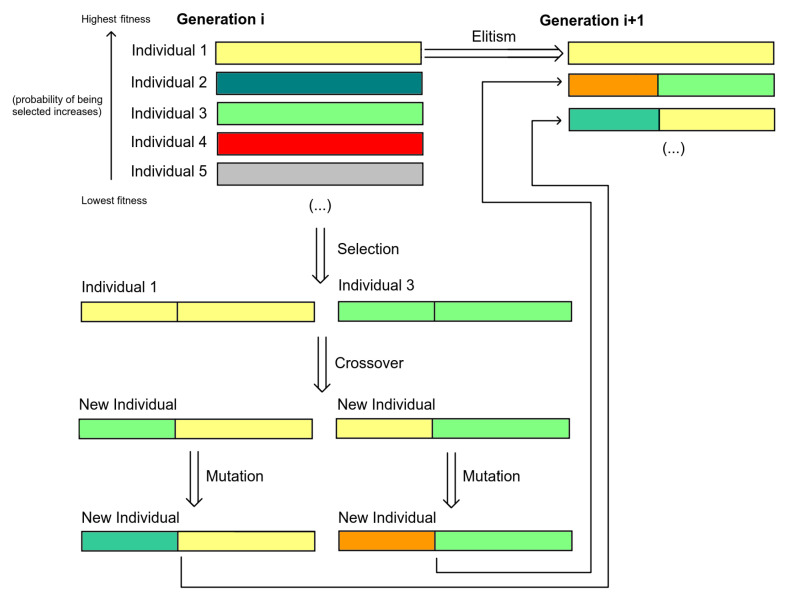
Genetic algorithm summary.

A visual summary of the genetic algorithm can be seen in Figure 8. The termination may vary from application to application. There are several possibilities, such as a specific criterion being achieved or a fixed number of generations reached.

In our case, each individual (candidate solution) was defined as a music piece. As already stated, a music piece is considered to be the gathering of several motifs (melody and harmony), their ordering, and tempo. Therefore, since each individual is a music piece, its genes are motifs. A property in each individual is the ordering of the motifs (genes).

Regarding the first step of the algorithm, the **Initializaion**, we decided to have a population of 100 individuals. The number of motifs generated per segment was the chosen number of individuals. Each individual was created by randomly choosing a motif from each segment. The initial order of the motifs was also randomly chosen.

To obtain each individual’s **Fitness**, a function composed of several different criteria were used:

- **Order Fitness**—The order of the motifs is evaluated according to how well it matches the probability distribution of intervals obtained from the histogram of angles from the whole sculpture. This measure ranges from 10 (perfect fit) to −10 (no fit).
- **Mode Definition Fitness**—For each motif, we evaluate how well the motif describes the respective mode. Each note has a weight according to its beat and its duration. Only the beat with the highest value is considered if the note is longer than one beat. If it is in the first beat, the weight is 4∗duration, the third 3∗duration, the second 2∗duration, and the fourth 1∗duration. The value for this measure is then calculated according to the type of note regarding the mode. Two concepts are used: if the note is characteristic of the mode and if the note is consonant. The characteristic intervals of a mode can be seen in Table 5. A note is considered consonant if the chord already includes that note or if the note does not create the interval m2 with any note from the chord. With this in mind, this fitness measure is then calculated as described in Table 6.
- **Range Fitness**—The range of the note pitches is also taken into account. The pitch should not be placed too low or too high. As such, a range was defined, where notes with a pitch outside of the range are disfavored. Here, the duration of the note and its placement regarding the bar are also taken into account. An acceptable range between 55 and 90 in MIDI was defined, and pitches that are not in the acceptable range are penalized −4.

With this fitness function, we evaluated how well the order represents the sculpture, how appropriate each motif is since the motif should represent the mode well enough, and if the notes’ pitch is in an acceptable range.

**Figure 9:**
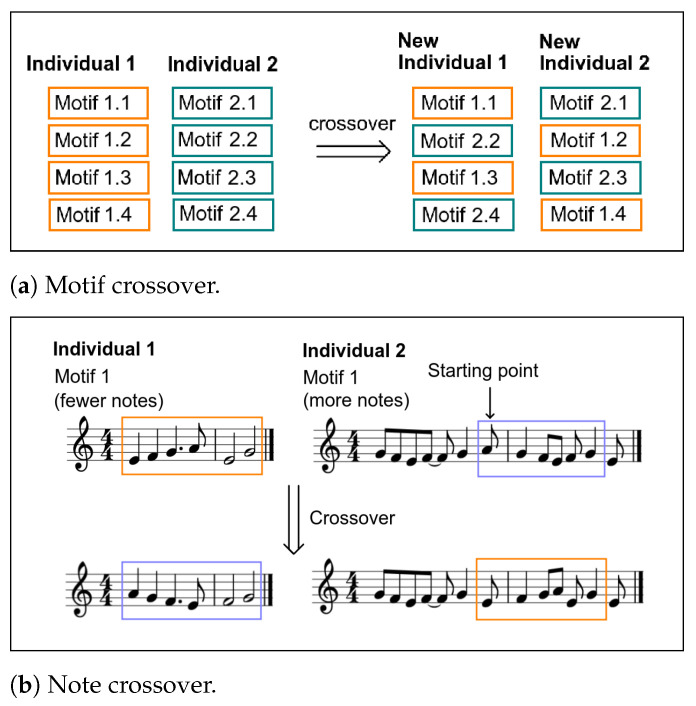
Crossover examples.

This is the stage where the genetic operators are used. Regarding **Elitism**, in each generation, 10% of the individuals with the highest fitness are directly passed on to the new generation. This guarantees some level of quality. After this, in the **Selection** stage, the individuals are selected as previously explained. Having a pair of selected individuals, there is an 80% probability of suffering **Crossover**. Two types of crossover were defined, both equally probable:

- **Motif crossover—**In this case, half of each individual’s motifs are randomly selected and crossed over. Since each motif came from a certain segment of the sculpture, the crossover occurs between motifs from the same segment. This approach was chosen to maintain a representative motif for each segment. An example of this type of crossover can be seen in Figure 9a. In this example, motifs 2 and 4 are randomly chosen to be crossed over.
- **Note crossover—**In this case, the note’s pitches are swapped. Having two motifs that came from the same segment (one of each individual), first, we calculate which one has more notes. Then, considering the difference in the number of notes between motifs, a crossover starting point is randomly chosen in the motif with more notes. This is the point from which the swap is made. There has to be sufficient space after the point for the smaller motifs’ pitches. The pitches from the smaller motif are traded with the pitches of the larger motif starting from the starting point. An example of this type of crossover can be seen in Figure 9b. Here, the motif with fewer notes is the one from individual 1, with six notes. Thus, the starting point in the motif with more notes (from individual 2) has to leave at least five more notes afterward. Since the longer motif has 12 notes, the starting point has to be randomly chosen between 1 (where the notes to be crossed over would be from 1 to 6) and 7 (where the notes to be crossed over would be from 7 to 12). In this example, the starting point is note 6. Thus, the notes crossed over in the longer motif are notes 6 to 11.

In the next stage, the individuals obtained have a probability of suffering **Mutation**. This involves altering the individual’s genes. Four types of mutation were used each with an independent probability:

- **Order mutation—**In this case, with a 10% probability for the individual, the order of the individual’s motifs is randomly altered.
- **“Tonify” mutation—**In this case, with 10% probability for each motif, notes that do not belong to the motif’s scale are corrected to the closest note found on the scale.
- **Inversion mutation—**With 5% probability for each motif, the inversion variation is applied, where the motif is mirrored, i.e., all the intervals are maintained, but their direction is the opposite, usually considering the first note as the reference.
- **Retrograde mutation—**With a 5% probability for each motif, the retrograde variation is applied, where the motif is reversed, i.e., the first note becomes the last and vice versa.

Considering the “tonify” mutation, one question may arise: why not merely limit the range of notes to the ones on each scale? This limitation would increase the fitness since, as explained before, notes that belong to the scale/mode are favored. However, much music that is generally considered good contains off-scale notes as long as they are placed correctly on the piece, and their duration is adequate. This case is taken into account when calculating the fitness value of the individual, where it is less penalized.

To terminate the algorithm, we decided to have a fixed number of generations. Regarding the final music piece, instead of obtaining the fittest individual in the final generation, our interest moved towards an approach that considers the *evolutionary process as artwork* [21].

In order to use this concept in our work, the fittest individual in each fixed number of generations were obtained. This number is defined according to the total number of generations. From our experiments, we decided to find the fittest individual in every third of the total number of generations. In the end, the individuals are joined and form the final music piece. This way, the music piece has a much better-defined structure, since motifs may be repeated from one individual to the other, although they may have suffered alterations from crossover or mutation. It is also interesting to consider that, when listening to the music, it becomes fitter as it goes by. We decided to terminate the algorithm once it reached 300 generations and join the fittest individual from every 100 generations.

### 3.5. Implementation

This project was implemented using Python [22] as the programming language, more specifically Python 3.6.8. Although the Python standard library is very extensive, other libraries were also used, since our work involves some specific domains.

First, the 3D Object domain mainly consists of meshes and texture. There are several different types of file formats, such as Stereolithography File (.stl), Wavefront 3D Object File (.obj), or Polygon Model File (.ply). We decided to focus our attention on Wavefront 3D Object files (.obj). If needed, the conversion from other formats to this one could be easily made. Regarding meshes, the library PyMesh [23] was used. This library is very useful since it provides an easy way to load a mesh, and to obtain some of its features. Unfortunately, it does not consider the mesh’s texture. As such, to have access to the texture, a file parser provided by PyGame [24] was used. The texture comes in an image file. As such, the Opencv-python [25] package was used to process it.

Considering the musical domain, MIDI (Musical Instrument Digital Interface) representation was used to represent the notes. To obtain the final music piece (in a lead sheet), first, the package MIDIUtil [26] was used to export the notes in MIDI format, then the software MuseScore [27] was used to obtain the final object, a lead sheet.

Considering all the processes between the sculpture and the music, some other packages were used for specific purposes. For handling arrays and some other functions, such as working with probabilities, package Numpy [28] was used, since this is a generally used package for scientific computing, with extensive documentation. For clustering, the package Scikit-learn [29] was used considering its capabilities regarding machine learning. Finally, for all the plotting purposes (from 2D histograms to more complex three-dimensional plotting of the sculptures), the package Matplotlib [30] was used.

## 4. Case Studies

Six 3D models of sculptures were used as our dataset. These were processed, and the final results were evaluated. The purpose of these cases is to give a clearer insight into our system’s behavior and verify if it accomplishes our objectives.

### 4.1. Dataset

The dataset used as input for our system has two sources: existing 3D models and 3D objects that were the result of capturing and processing existing sculptures by the author. These sculptures were selected due to their variety in shape and texture and, therefore, help us to better evaluate our system’s capability of generalization. There were limitations regarding both the availability of 3D models online and the sculptures to be scanned. The end result were three distinct sculptures by the same author and three other sculptures with different authors and periods.

**Figure 10:**
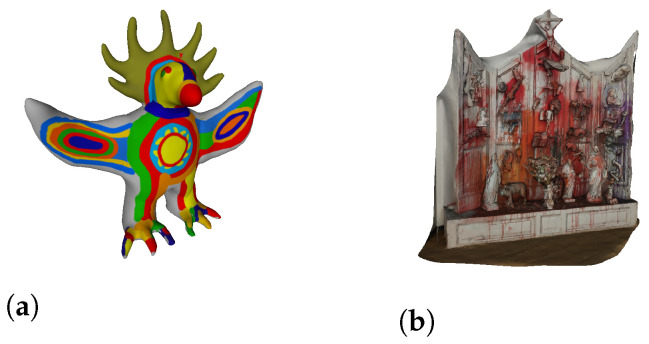
Two models used from Sketchfab [31]. (**a**) Sun God (https://skfb.ly/6JTCB (accessed on 2 December 2021)); (**b**) Tir (Shooting Altar) (https://skfb.ly/6IFJC (accessed on 2 December 2021)).

For existing 3D models, many models can be found online in several locations. We used the platform Sketchfab [31], from which two models were retrieved (Figure 10).

**Figure 11:**
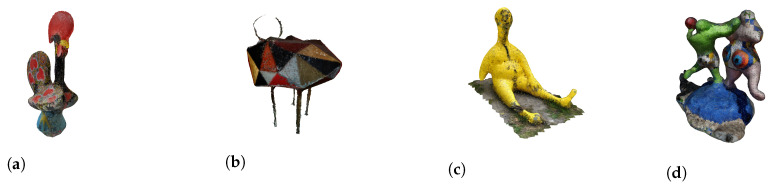
3d Objects resulting from the capture and processing of sculptures. (**a**) Galo de Barcelos (Owned by the author. Available at https://skfb.ly/6Nzzv (accessed on 2 December 2021)); (**b**) Unkown Sculpture (Location: Museu Nacional de Arte Contemporânea do Chiado, Lisboa. Available at https://skfb.ly/6NzAH (accessed on 2 December 2021)); (**c**) Sr. Estúpido by Robert Panda (Location: Telheiras, Lisboa. Available at https://skfb.ly/6NzzE (accessed on 2 December 2021)); (**d**) Les Baigneuses by Niki de Saint Phalle (Location: Centro Cultural de Belém, Lisboa. Available at https://skfb.ly/6NzAz (accessed on 2 December 2021)).

For the acquired models, the authors used the device *Lenovo Phab 2 Pro* for the acquisition. The result of this capture is a point cloud. As such, some processing was needed to convert it into a polygonal mesh. For this purpose, the open-source software Meshlab [32] was used. Four sculptures were captured and processed. Thus, four 3D objects were used from this second source. The final result of the process (and the input for our system) for the four sculptures can be seen in Figure 11. One important remark is that 3D Objects found in Figure 11a,c,d were processed using the Poisson Surface reconstruction approach. The one found on Figure 11b was processed using the Ball Pivoting reconstruction approach.

All the 3D models of the sculptures and respective interpretations of the composed music are available at http://web.tecnico.ulisboa.pt/ist181444/ (accessed on 2 December 2021).

### 4.2. Evaluation

To evaluate our system, our objectives were considered. With these in mind, the evaluation aimed at verifying if the music pieces composed are associated with the sculpture and if the music is considered aesthetically pleasing. Although one of our goals was to make a system that acts creatively and generates creative products, this aspect was not evaluated directly. Instead, we considered that the evaluation of the other two goals would lead to this goal’s evaluation. It is relevant to stress that only the Genetic Composition was evaluated. We decided to use online surveys to perform the evaluation.

The final output of our system is a lead sheet for each sculpture. However, to be able to evaluate the final compositions, an audio file needed to be generated. Due to time constraints, it was not possible to record the composed pieces with musicians. Instead, for each music piece, three interpretations were created using virtual instruments.

The first interpretation can be considered as closer to Jazz. The instruments used were a piano and a double-bass. A drum set was not used in this interpretation since the virtual sound of a drum set in Jazz style does not sound realistic. The second interpretation can be seen as closer to the Rock Genre. In this case, a piano, electric bass, and drum set were used. The third interpretation is difficult to define stylistically. This is a more electronic interpretation, yet it may also be seen in the Space Rock genre. For this one, two Synthesizers, one electric guitar, one electric bass, and a drum set were used. All interpretations were created considering the common rhythmic patterns of the style for the accompaniment and the drum rhythms.

As already stated, the final compositions were evaluated using online surveys. Two surveys were made, each with three compositions, and, for each composition, the three interpretations. The survey starts off by asking for the age group (<18; 18–29; 30–45; 46–60; >60) and musical knowledge (None, Basic, Intermediate, and Advanced). Regarding the musical knowledge, each degree had either a small description or some examples for more coherent answers:

- “None—only listener”.
- “Basic—Example: novice in an instrument, starting to take music lessons, played instrument when younger”.
- “Intermediate—Example: Currently taking classes, attending a music course, has some knowledge on ear training”.
- “Advanced—Example: Have a music degree, professional musician with music theory background”.

Afterwards, for each musical composition, an excerpt from each interpretation was shown and ranked by the participant. Considering each participant’s rank, each listened to the full preferred version. The following questions were then made:

- “Do you consider this to be music?” Yes or No.
- “How would you rate this music?” 1 (Terrible music)—5 (Really good music).
- “How would you describe this music? (You may choose several options)” Happy; Exciting; Smooth; Mellow; Sad; Rough; Bitter; Harsh; Aggressive; Boring; and Other.

With the first two questions, we aimed at evaluating the music itself and, with the third, to obtain a more subjective opinion on the music.

The survey then moved to the part related to the sculpture. First, the 3D model of the sculpture was shown without making any reference to it being the inspiration for the musical composition. Then, we asked the question (with the following possible answers): “How would you describe this sculpture? (You may choose several options)” Happy; Exciting; Smooth; Mellow; Sad; Rough; Bitter; Harsh; Aggressive; Boring; and Other.

This question has the same possible answers as the one regarding the music’s description. With this question, it was our intention to make the audience carefully look at the sculpture and obtain a general description of the sculpture.

Finally, the relation between music and sculpture was evaluated. Here, the participant was shown how he/she previously described the music and informed that the music was composed having the previous sculpture as inspiration. Then, we asked: “Do you think they are related?” 1 (Not at all)—5 (Totally). The final questions for each composition is to ask the music’s rating and if the audience thinks the music is related to the sculpture (same as before) for the least preferred interpretation. With these two, we aimed at evaluating the importance of the interpretation and establishing a floor for the results.

The division of musical compositions/sculpture per survey was intentional. For each, one sculpture obtained online and two captured and processed by us were chosen. As such, the division is the following (sculptures in the presented order):

- **Survey 1—**Tir (Shooting Altar) (Figure 10b), Les Baigneuses (Figure 11d), and Unkown Sculpture (Figure 11b).
- **Survey 2—**Sr. Estúpido (Figure 11c), Galo de Barcelos (Figure 11a), and Sun God (Figure 10a).

For Survey 1, answers were collected from a total of 62 participants, and for Survey 2, for a total of 36 participants. Below, the most relevant data obtained are analyzed.

### 4.3. Results and Discussion

**Figure 12:**
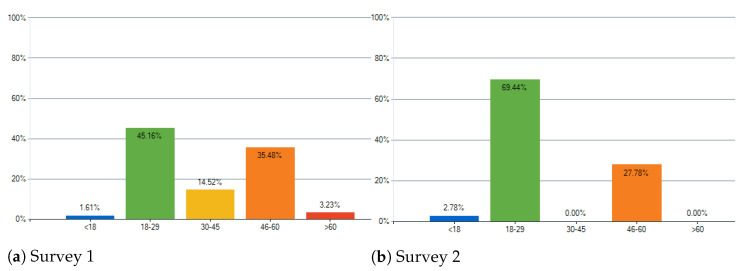
What is your age group?

**Figure 13:**
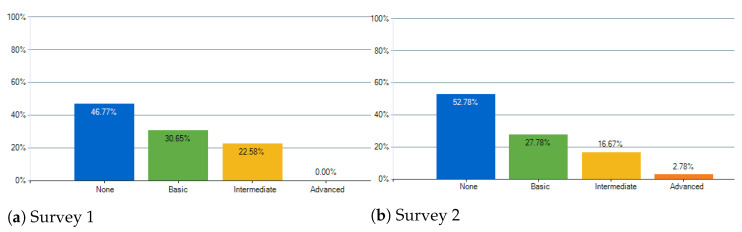
How would you describe your musical knowledge?

Starting with the age groups, the two largest groups for both surveys were 18–29 and 46–60. The obtained answers can be seen in Figure 12. About the musical knowledge, the results were the expected, where there are fewer for deeper Musical Knowledge. The obtained answers can be seen in Figure 13.

Using the relevant information about the participants, regarding the ranking of interpretation/version, the statistics relative to the answers are presented in Table 7. Since each participant ranked the three versions, the mode is the most relevant measure, i.e., the value most often chosen. From this, we concluded that the most preferred version was the first (closer to Jazz). However, considering the mean and standard deviation measures, it is possible to state that the difference among interpretations is not much.

Considering the first question regarding the preferred interpretation (Do you consider this to be music?), the statistics based on the results can be seen in Table 8. Regarding the results, for all compositions, more than 90% of the participants considered the preferred interpretation to be music.

Furthermore, regarding the responses on the preferred music’s rating, the statistics obtained from the results can be found in Table 9.

For all the musical compositions, on a scale of 1 (lowest) to 5 (highest), the mode was 4, i.e., the rating most often encountered was 4. From the median, we concluded that, for five of the six compositions, at least 50% of the participants ranked 4 or higher. The statistics for the same question, but regarding the least preferred version, can be seen in Table 10. The results were lower, as expected. The median, instead of 4, was 3, meaning that more than 50% of the participants still rated the least preferred interpretation as 3 or higher. The mode was one step below the preferred version. The data for the rating of the least preferred version regarding the sculpture Galo de Barcelos was not collected due to an error in the process of survey making.

Considering the sculpture–music association, the participants were asked to select descriptors among a given set that better describe the preferred interpretation and the sculpture (in separate). The descriptors used were: Happy, Exciting, Smooth, Mellow, Sad; Rough, Bitter, Harsh, Aggressive, Boring, and Other. To evaluate the association, we verified whether the descriptors assigned to the sculpture corresponded with those of the music. The histogram of the descriptors was made, and for each sculpture–music pair, the Bhattacharyya Coefficient [33] was calculated (Table 11) as a measure of their similarity.

The statistics for the responses regarding the rating of the sculpture–music relationship can be seen in Table 12 for the preferred version and in Table 13 for the least preferred one.

Two special cases were found concerning the results: one case in which the results were very low and one in which the opinions regarding the descriptions were very widespread.

The first is the *Tir (Shooting Altar)* sculpture (Figure 10b), where its semantics leads to a drastically different perspective from the one obtained through our approach. In this case, the description given by the participants for the sculpture was distant from the composition, as evidenced by the Bhattacharyya Coefficient in Table 11. The composition was not considered related to the sculpture, can be confirmed by the statistics in Table 12, where the mode measure for the relation rating was 1 and the median 2. The results were similar for the least preferred version (Table 13).

Looking closer at the sculpture (Figure 10b), one can see that it is quite complex. It is a white altar, an object from the religious world with strange figures and objects in it, with bright red in some parts that, in this case, can be related to blood. For our system, these colors (white and bright red) were related to brighter modes, which do not translate the semantics of this sculpture. As already stated, it was not our intention to retrieve the sculptures semantics, as such, these results were understandable.

The second is the *Unknown Sculpture* (Figure 11b), where there was much variation on how humans perceived the sculpture. Considering that the participants were describing artwork, it is reasonable to have disagreement in some cases. This case happened to be one where each of the participant’s perspective varied greatly. The Bhattacharyya Coefficient is high because both the music and sculpture descriptions were ambiguous without any characteristic descriptor.

From the statistics of the results for the preferred version, seen in Table 12, the previous hypothesis can be confirmed. The mean was 2.9, the second lowest, while the mode and median were 3. Regarding the least preferred version, the mode was 1 and the median 2. Curiously, the mean value was higher than the preferred one. These results, once again, prove that there was not much agreement regarding this sculpture, since the results for the relation rating were widely dispersed.

For the typical case, which was found in the remaining four of the six music/sculpture pairs, most people related the music to the sculpture. Regarding the description, there was a clear connection between the sculpture and the music as seen by the Bhattacharyya Coefficient in Table 11. For the sculpture *Sr. Estúpido*, the median for the relation rating for the preferred interpretation was 5, while for *Les Baigneuses* and *Sungod* 4 and for *Galo de Barcelos* was 3. For the least preferred, the median for the rating was 3 for the four cases.

## 5. Conclusions and Future Work

We presented a system that aims at composing music inspired by 3D models of sculptures. To build this, an existing model for inspiration [5] was used, involving different human senses, and applied to a pair of domains that, to the best of our knowledge, have never been explored before. One of the unique aspects of our system is the chosen mapping between the two different domains, which is the base for the applied inspirational model.

Only the *Genetic* Composition was considered for evaluation. This evaluation was based on answers from online surveys, which focused on assessing two of our goals: the quality of the musical composition and the sculpture–music association. The musical compositions were well rated, thus, providing evidence of their quality. Regarding the sculpture–music association, despite two exceptional cases, the results provide evidence of this association.

We stress that this paper describes one possible approach led by the aesthetics of the authors among an infinite number of other possible approaches. One alternative approach would be to relate the two different domains using an isomorphic mapping between two different mathematical lattices [34]. Regarding possibilities closer to our work, some were tested during the process; however, these are a small fraction compared to what could be explored.

Our work was also limited regarding the musical concepts and elements used; therefore, other approaches could be explored. For example, in the music realm, a more general approach could be implemented, considering not only modal harmony but also tonal or atonal. As for the association, other possibilities could be tested. Although the shape–harmony and texture–melody associations were used in our work, other possibilities could be explored and even available for a user to choose.

Other approaches to the genetic algorithm could also be investigated. One possibility would be to give more freedom to the algorithm by not limiting the crossover between motifs generated from the same segment of the sculpture. This could be compensated by using a fitness measure that would favor individuals where all the segments are represented. Another aspect that could be worked on is evaluation since only one of the three output compositions was evaluated. These three versions could be assessed to verify, for example, which one is preferred and which one is considered to be more related to the sculpture. In addition to this, having human interpretations of the compositions could significantly improve the evaluation. The quality of music played by real musicians is different from interpretations generated using a computer.

## Figures and Tables

**Table 1:** Common interval names and abbreviations.

Number of Semitones	Common Interval Name	Abbreviation
0	Perfect Unison	P1
1	Minor Second	m2
2	Major Second	M2
3	Minor Third	m3
4	Major Third	M3
5	Perfect Fourth	P4
6	Augmented Fourth	A4
	Tritone	TT
	Diminished Fifth	d5
7	Perfect Fifth	P5
8	Minor Sixth	m6
9	Major Sixth	M6
10	Minor Seventh	m7
11	Major Seventh	M7
12	Perfect Octave	P8

**Table 2:** Mapping from angles to musical intervals.

Angles (Degrees)	0°–150°	15°–30°	30°–45°	45°–60°	60°–75°	75°–90°	90°–105°	105°–120°	120°–135°	135°–150°	150°–165°	165°–180°
**Interval**	M2	M3	m3	P1	M6	m6	P5	P4	m7	m2	M7	TT

**Table 3:** Mean autocorrelation and most probable rhythmic value mapping.

Mean Autocorrelation	0–0.05	0.05–0.1	0.1–0.4	0.4–0.7	0.7–1
Most Probable Rhythmic Value	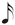	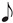	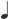	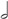	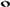

**Table 4:** Obtaining each rhythmic value using a normal distribution.

Most Probable Rhythmic Value	Value Obtained Using Normal Distribution (*x*)	Probability	Rhythmic Value Chosen
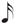	−1 < x < 1	68.3%	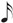
	−2 < *x* < −1 or 1 < *x* < 2	27.1%	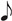
	*x* < −2 or *x* > 2	4.6%	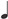
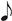	−1 < *x* < 1	68.3%	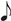
	*x* < −1	15.85%	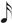
	*x* > 1	15.85%	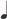
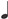	−1 < *x* < 1	68.3%	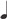
	*x* < −1	15.85%	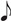
	*x* > 1	15.85%	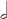
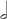	−1 < *x* < 1	68.3%	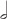
	*x* < −1	15.85%	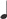
	*x* > 1	15.85%	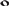
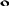	−1 < *x* < 1	68.3%	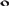
	−2 < *x* < −1 or 1 < *x* < 2	27.1%	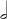
	*x* < −2 or *x* > 2	4.6%	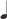

**Table 5:** Summary of the major scale modes (characteristics notes in bold).

Degree of Major Scale	Mode	Intervals	Chord (Tetrad)
I	Ionian	P1 M2 M3 **P4** P5 M6 **M7**	IΔ
II	Dorian	P1 M2 m3 P4 P5 **M6** m7	IIm7
III	Phrygian	P1 **m2** m3 P4 P5 m6 m7	IIIm7
IV	Lydian	P1 M2 M3 **A4** P5 M6 M7	IVΔ
V	Mixolydian	P1 M2 M3 P4 P5 M6 **m7**	V7
VI	Aeolian	P1 M2 m3 P4 P5 **m6** m7	VIm7
VII	Locrian	P1 m2 m3 P4 **d5** m6 m7	VIIm7♭5

**Table 6:** Mode definition fitness measure for different types of notes.

			weight <=1	weight >1
**Note** **in** **Mode**	Characteristic	Consonant	2*weight	
		Dissonant	2*weight	−weight
	Not Characteristic	Consonant	weight	
		Dissonant	weight	−weight
**Note not in Mode**			weight	−weight

**Table 7:** Which version do you prefer?—Statistics.

Question		Measure	Tir (Shooting Altar)	Les Baigneuses	Unkown	Sr. Estúpido	Galo de Barcelos	Sun God
Which version do you prefer?	Music 1	Mean Median Mode Std. Dev.	2 2 3 0.9	1.7 1.5 1 0.8	1.5 1 1 0.7	1.9 2 1 0.9	1.6 1 1 0.8	1.8 2 1 0.8
	Music 2	Mean Median Mode Std. Dev.	1.7 1.5 1 0.8	2 2 2 0.8	2 2 2 0.7	1.9 2 1 0.8	2.1 2 2;3 0.8	1.8 2 1 0.8
	Music 3	Mean Median Mode Std. Dev.	2.3 2 2;3 0.7	2.2 2 3 0.8	2.5 3 3 0.7	2.2 2 2 0.7	2.3 2 3 0.8	2.4 3 3 0.7

**Table 8:** Do you consider this to be music?—Percentage.

Question	Answer	Tir (Shooting Altar)	Les Baigneuses	Unkown	Sr. Estúpido	Galo de Barcelos	Sun God
Do you consider this to be music?	Yes	95.16%	96.77%	90.32%	100%	100%	100%
	No	4.84%	3.23%	9.68%	0%	0%	0%

**Table 9:** How would you rate this music? (Preferred)—Statistics.

Question	Measure	Tir (Shooting Altar)	Les Baigneuses	Unkown	Sr. Estúpido	Galo de Barcelos	Sun God
How would you rate this music?	Mean Median Mode Std. Dev.	3.4 3.5 4 0.8	3.6 4 4 0.9	3.1 3 4 1.2	3.8 4 4 0.6	3.8 4 4 0.7	3.9 4 4 0.7

**Table 10:** How would you rate this music? (Least Preferred)—Statistics.

Question	Measure	Tir (Shooting Altar)	Les Baigneuses	Unkown	Sr. Estúpido	Galo de Barcelos	Sun God
How would you rate this music?	Mean Median Mode Std. Dev.	2.7 3 3 1	2.7 3 3 0.9	2.5 3 3 1.1	3.1 3 3 0.8	-	2.9 3 3 1.1

**Table 11:** Bhattacharyya coefficient for the music/sculpture descriptions.

Sculpture	Tir (Shooting *Altar)*	Unknown *Sculpture*	Sungod	Les Baigneuses	Galo de Barcelos	Sr. Estúpido
Bhattacharyya Coefficient	0.4981	0.8963	0.8539	0.8848	0.9426	0.8491

**Table 12:** Do you think the are related? (Preferred)—Statistics.

Question	Measure	Tir (Shooting Altar)	Les Baigneuses	Unkown	Sr. Estúpido	Galo de Barcelos	Sun God
Do you think they are related?	Mean Median Mode Std. Dev.	2 2 1 1.2	3.4 4 4 0.9	2.9 3 3 1.1	3.5 5 5 1.3	3.2 3 3 1.2	3.4 3.5 4; 5 1.3

**Table 13:** Do you think this version is related to the sculpture? (Least Preferred)—Statistics.

Question	Measure	Tir (Shooting Altar)	Les Baigneuses	Unkown	Sr. Estúpido	Galo de Barcelos	Sun God
Do you think this version is related to the sculpture?	Mean Median Mode Std. Dev.	2 2 1 1	2.8 3 4 1.3	3.5 2 1 1.3	3.1 3 2;3 1.3	3 3 3 1.2	3 3 4 1.2

## Data Availability

All the 3D models of the sculptures and respective interpretations of the composed music are available at http://web.tecnico.ulisboa.pt/ist181444/ (accessed on 2 December 2021).
